# A comparative survey of genetic diversity among a set of Caricaceae accessions using microsatellite markers

**DOI:** 10.1186/2193-1801-2-345

**Published:** 2013-07-26

**Authors:** Samik Sengupta, Basabdatta Das, Manoj Prasad, Pinaki Acharyya, Tapas Kumar Ghose

**Affiliations:** Division of Plant Biology, Bose Institute, Main Campus, 93/1 A.P.C. Road, Kolkata, West Bengal 700009 India; Department of Horticulture, Institute of Agricultural Science, University of Calcutta, 35, Balligunge Circular Road, Kolkata, West Bengal 700029 India; National Institute of Plant Genome Research (NIPGR), Aruna Asaf Ali Marg, New Delhi, 110067 India

**Keywords:** Caricaceae, Carica papaya, Genetic diversity, SSR

## Abstract

A preliminary survey of genetic diversity among 34 commercially popular *Carica papaya* cultivars from India and abroad, 6 accessions of *Vasconcellea* species and 1 accession of *Jacaratia spinosa*, was done using 20 simple sequence repeat (SSR) markers. The SSR profiles were used to find out total number of alleles, null and rare alleles, Polymorphism Information Content (PIC) values and to calculate similarity matrix using Jaccard’s coefficient. The subsequent dendrogram was made by unweighted pair-group method of arithmetic average (UPGMA) and neighbor-joining method. Based on these parameters a comparison was made between the Indian papaya cultivars and the rest of the accessions. All the markers showed polymorphism and a total of 140 alleles were identified. The average number of alleles was 7 alleles/locus. Categorically the *Vasconcellea* and *Jacaratia* species had 54 alleles, the 7 non-Indian *Carica papaya* accessions had 70 and the 27 Indian accessions had 102 alleles. The average PIC value was 0.735 per marker. A total of 37 rare alleles were identified. *Jacaratia spinosa* had 17 rare alleles. Nineteen null alleles were detected among the *Carica papaya* accessions. A *Carica papaya* accession from South Africa, Hortus Gold had 5 null alleles. The genetic similarity among the accessions ranged from 7% to 67%. In the dendrogram, the *Vasconcellea and Jacaratia spinosa* accessions separated as a distinct cluster from the rest of the *Carica papaya* accessions. The study indicated that the accessions of Indian *Carica papaya* cultivars included in this survey are genetically more diverse than the non-Indian *Carica papaya* cultivars.

## Background

The family Caricaceae consists of six genera of herbaceous, shrubby or arborescent dicotyledonous plants having a common phylogenetic origin with Brassicaceae, and consequently with the completely sequenced model plant *Arabidopsis* (Bremer et al. 1998 Rodman et al. 1996). From the perspective of human consumption *Carica* and *Vasconcellea* are the two most important genera within Caricaceae (Badillo 1993 and 2000). *Vasconcellea* grows at a height of 1000 m and higher, above sea level, mostly in wild humid forests, but are also semi-domesticated or tolerated in local gardens in the highlands of South America. The only *Vasconcellea* species cultivated in an intensive way, although only in Ecuador and New Zealand is the cultivar Babaco (*V. × heilbornii*; Villarreal et al. ‘Babaco’, Villarreal et al. Villarreal et al. 2003). On the other hand *Carica papaya* (commonly known as papaya) originated and was subsequently domesticated in Central America and has hundreds of cultivars spread over 50 countries around the world (Purseglove 1974; Storey 1969; Kulsekaran 1984; Nakasone and Paull, 1998; FAO 2009). Wide range of germplasm variability, rapid development and copious production of seeds have made papaya a potentially valuable fruit tree model crop for genomic and genetic diversity analysis (Liu et al. 2004; Yu et al. 2008). Some 2.5 billion kilograms of papaya are produced annually in India in the states of Andhra Pradesh, Assam, Bihar, Gujarat, Karnataka, Maharashtra, Manipur, Meghalaya, Orissa, Tamil Nadu, Uttar Pradesh and West Bengal. It is eaten fresh or cooked and is processed into pickles, jams, candies, fruit drinks and juices. Papain, an enzyme purified from papaya latex, is extracted for export. The enzyme is used in medicine and textile industries, breweries, leather processing and in meat tenderizing. Ram et al. (1985) classified the popular Indian papaya cultivars like Coorg Honey Dew, Washington, CO1, CO2, PusaDwarf, Surya etc. into various groups like primitive genotypes, traditional genotypes, minor genotypes, local adaptive genotypes and principal genotypes. The germplasms of *Carica* show considerable phenotypic variation in plant stature, length of juvenile period, fruit size, fruit shape, flesh color, flavor and sweetness, stamen carpelloidy, and carpel abortion (Drew et al. 1998; Manshardt and Wenslaff, 1989). Although India has a vast array of indigenous papaya cultivars (Ram 2005), reports of systematic collection, conservation, documentation and evaluation of different germplasms and study of their inherent genetic diversity are limited.

Analysis of Simple Sequence Repeat (SSR) polymorphism is a popular molecular tool for surveying genetic diversity (Xiao et al. 1996). There are plenty of reports on analysis of Caricaceae genotypes using SSR markers. Asudi et al. (2010) collected 42 papaya germplasm from Coast, Nyanza, Western, Rift Valley, Eastern and Central provinces of Kenya, characterized them morphologically and also assessed their genetic diversity using 7 SSR markers. They found that number of alleles across the seven loci ranged from 8 to 18 with an average of 11.93 alleles/locus. The polymorphism information content (PIC) varied from 0.75 to 0.852 with an average of 0.81. Oliveira et al. (2010a and 2010b) identified 20 polymorphic microsatellite primers which they used for marker assisted selection of 83 papaya lines and identified an average of 3.18 alleles per primer. Kyndt et al. (2006) analysed a set of 103 *Vasconcellea* accessions and some individuals of the related genera *Carica* and *Jacaratia* with the help of 10 chloroplast and 9 nuclear SSR markers. Six of the chloroplast and seven nuclear SSRs showed polymorphism. Other molecular markers like random amplified polymorphic DNA (RAPD) (Sondur et al. 1996) and amplified fragment length polymorphism (AFLP) (Ma et al. 2004) were used to construct papaya genetic maps. In 2001, Ming et al. (2001) reported the construction of a papaya bacterial artificial chromosome (BAC) library which contained 39,168 clones from the Hawaiian papaya cultivar “Sun Up”. Chun et al. (2006) have analyzed the microsatellite content, repeat element composition and protein-coding regions from the BAC library of the hermaphrodite papaya cultivar SunUp. Later, Eustice et al. (2008) mined 28.1 Mb of BAC end sequences, 5.8 Mb of complementary DNA, and 1.6 Mb of random genomic sequences and identified 938 SSR markers. A major study on papaya diversity in India was done by Singh et al. (2006). They collected and studied 21 accessions of papaya comprising of Indian and exotic cultivars and identified 2 excellent varieties ‘Pusa Delicious’ and ‘CO 7′ with good growth attributes and physico-chemical characters of the fruits. Given the number of papaya cultivars available in India, more investigations into papaya genetic diversity are required to facilitate unambiguous identification of the various germplasms and their protection under the trade related intellectual property rights (TRIPS) of the World Trade Organization (WTO). This study surveyed the inherent genetic diversity of 34 commercially popular *Carica papaya* cultivars and seven species of Caricaceae genotypes using 20 SSR markers. The SSR profiles were used for comparisons between the accessions of Indian and non-Indian papaya cultivars. Statistical calculations were used for grouping the accessions into various clusters in order to unambiguously identify and to establish a relationship among the accessions.

## Results

### Analysis of SSR profiles

#### Number of alleles

**Figure 1:**
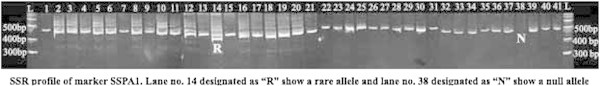
**Gel image showing the presence of rare and null allele.**

Table 1 summarizes the analyses of SSR profiles of 41 accessions using 20 SSR markers. Figure 1 shows a SSR profile depicting rare and null allele. The minimum and maximum molecular weight among the alleles, number of rare alleles, number of null alleles, total number of alleles and PIC values for each marker are given in table. The alleles were scored based on the molecular weight. The reference molecular weight for each marker (as in the accession SunUp) is also shown in Table 1. All the markers showed polymorphism and a total of 140 alleles were identified from the experimental set of Caricaceae accessions. The number of alleles ranged from 2 in SP4 and SP8 to 11 in SP1, SSPA1, SSPA5 and SSPA8. The average number of alleles was 7 alleles/locus. The average number of alleles for markers amplifying both dinucleotide and trinucleotide repeats, was also 7. Categorically the total number of alleles for 6 *Vasconcellea* and 1 *Jacaratia* accessions was 54 with an average of 2.7 alleles/locus. The smallest number of alleles identified was 1 each in the markers SP4 and SP8. The highest number of alleles in this category was 5, detected from the profile of the marker SSPA8. The average number of alleles for markers amplifying dinucleotide repeats (2.9 alleles/locus) was more than that for trinucleotide repeats (2.5 alleles/locus). The total number of alleles for the 7 non-Indian *Carica papaya* accessions was 70 with an average of 3.5 alleles/locus. The smallest number of alleles identified was 2 each in the profiles of the markers SP2, SP4, SP7, SP8, SP10 and SSPA2. The highest number of alleles in this category was 6, identified from the profile of the markers SP1 and SSPA5. The average number of alleles from markers amplifying dinucleotide repeats (3.57 alleles/locus) was more than that for trinucleotide repeats (3.33 alleles/locus). The total number of alleles for the 27 Indian *Carica papaya* accessions was 102 with an average of 5.1 alleles/locus. The smallest number of alleles identified was 2 in SSR profiles of the marker SP8. The highest number of alleles in this category was 9 from the profiles of the markers SP1, SP2 and SSPA1. Contrary to the others, in this category, the average number of alleles from the markers amplifying dinucleotide repeats (4.93 alleles/locus) was less than that for trinucleotide repeats (5.5 alleles/locus).

**Table 1:** **Minimum and maximum molecular weight among the alleles, Rare alleles (R), null alleles (N), alleles and PIC values for each marker**

Marker	Min MW	Max MW	Sun Up MW	R	N	Number of alleles				PIC values			
						Total	V&J	FA	IA	Total	V&J	FA	IA
SP1	415.405	424.341	489	1	1	11	3	6	9	0.919	0.571	0.836	0.894
SP2	125.854	543.174	678	4	3	10	3	2	9	0.929	0.448	0.632	0.941
SP3	341.25	445.714	656	2	0	9	3	5	7	0.909	0.612	0.775	0.877
SP4	165	170	594	0	0	2	1	2	2	0.499	0	0.489	0.482
SP5	246.263	270.884	611	1	2	7	3	4	5	0.810	0.571	0.795	0.796
SP6	385	409.062	698	1	1	6	3	3	4	0.728	0.448	0.448	0.684
SP7	168.988	176.02941	717	1	1	6	3	2	3	0.668	0.571	0.469	0.559
SP8	127.49	135.64	603	0	0	2	1	2	2	0.492	0	0.489	0.384
SP9	180.976	196.77975	764	2	0	5	3	3	3	0.696	0.571	0.571	0.658
SP10	232.555	485.333	701	1	1	6	3	2	4	0.715	0.448	0.591	0.622
SSPA1	425.321	488.296	374	1	1	11	3	5	9	0.884	0.612	0.734	0.862
SSPA2	272.5	389.285	348	2	1	6	3	2	3	0.581	0.571	0.244	0.448
SSPA3	159.372	181.415	156	2	0	7	4	3	4	0.652	0.591	0.571	0.614
SSPA4	195.035	206.143	203	1	0	6	3	4	5	0.745	0.224	0.693	0.652
SSPA5	402.068	482.090	438	6	0	11	2	6	7	0.862	0.244	0.816	0.806
SSPA6	117.426	125.800	124	1	0	5	2	3	4	0.658	0.244	0.612	0.529
SSPA7	267.75	615.517	290	2	2	7	2	3	6	0.731	0.244	0.571	0.661
SSPA8	264.102	363.545	326	5	2	11	5	5	8	0.861	0.775	0.816	0.817
SSPA9	89.166	110.379	107	2	1	5	2	4	3	0.589	0.244	0.612	0.524
SSPA10	195.238	215.25	202	2	3	7	2	4	5	0.765	0.244	0.734	0.696
**Total SUM**				37	19	140	54	70	102	14.701	8.244	12.510	13.517
**Average AVERAGE**				1.85	0.95	7	2.7	3.5	5.1	0.735	0.412	0.625	0.675

*MinMW* Minimum molecular weight of the alleles in that locus, *Max MW* Maximum molecular weight of the alleles in that locus, *SunUp MW*,

Molecular weight of the allele obtained from the reference genotype SunUp, *V*& *J* accessions of *Vasconcellea* and *Jacaratia, FA* foreign *Carica papaya* accessions, *IA* Indian *Carica papaya* accessions.

### Polymorphism Information Content (PIC) values

The PIC values, which denote allelic diversity and frequency among germplasms, had an average value of 0.735/marker. The range of PIC values was 0.492 in SP8 to 0.929 in SP2. Categorically average PIC value for the *Vasconcellea* accessions was 0.412/marker with a range of 0.244 for the markers SSPA5, SSPA6, SSPA7, SSPA9 and SSPA10 to 0.775 for the marker SSPA8. For the non-Indian papaya accessions the average PIC value was 0.625/marker and range of PIC values was 0.245 for the marker SSPA2 to 0.837 for SP1. The Indian papaya accessions had an average PIC value of 0.676/marker. The range of PIC values was 0.384 for the marker SP8 to 0.941 for SP2. From the PIC values it was evident that the allelic diversity was the highest among the Indian papaya accessions. The average PIC value of the markers amplifying dinucleotide repeats was 0.727. The same for trinucleotide repeat containing markers was 0.753. This trend was maintained in all the other categories except in case of the *Vasconcellea* accessions where the average PIC value for the trinucleotide repeat amplifying markers was less than average PIC value for the dinucleotide repeat amplifying markers. Analysis of Variance test (ANOVA test) was done to test whether the means of the PIC values for the total set of germplasms, and for the three categories (Indian and non Indian *Carica papaya* accessions and the accessions of *Vasconcellea* species and *Jacaratia spinosa*) were significantly different or not. The result of the test is tabulated in Table 2. From the ANOVA test it was found that the observed F value exceeded the tabulated F value at 1%. Hence the null hypothesis that the means PIC values of the three categories i.e. Indian and non-Indian *Carica papaya* accessions and the accessions of *Vasconcellea* species and *Jacaratia spinosa* were same, was rejected. From the calculated critical difference it was observed that the average PIC values were significantly different when comparison was made between *Vasconcellea* sp. and *Jacaratia spinosa* accession and Indian accessions. The same applied when a comparison was made between *Vasconcellea* sp. and *Jacaratia spinosa* accession and non-Indian accessions. But the PIC values between the accessions of Indian and non-Indian *Carica papaya* accessions were not significantly different.

**Table 2:** **Analysis of variance table for polymorphism information content values**

Source of variation	Sum of squares	Degrees of freedom	Mean sum of squares	F Value	
				Observed	Tabulaed
**Between markers**	30.5023	19	1.6054	44.5394	1 (at 5%)
**Within markers (error)**	2.1626	60	0.036		1 (at 1%)

### Rare alleles

As per the definition of rare alleles by Jain et al. (2004) a total of 37 rare alleles were identified from 18 polymorphic loci with an average of 1.85 rare alleles per loci. A rare allele is shown in the gel image given in Figure 1. Average rare alleles from the dinucleotide repeat amplifying markers was 2 and that from the trinucleotide repeat amplifying marker was 1.5. The highest number of rare alleles (8 rare alleles) was identified in the profile of SSPA5 followed by SSPA 8 (7 rare alleles) and SSPA 2 (5 rare alleles). Among the Caricaceae accessions, *Jacaratia spinosa* had the maximum number of rare alleles (17 rare alleles) almost one for each of the 20 markers used. Four *Vasconcellea* accessions shared amongst themselves 5 rare alleles. *Vasconcellea quercifolia* had 2 and *V. pubescens, V. goudotiana and V. microcarpa* had 1 rare allele each. Among the *Carica papaya* cultivars, five non-Indian accessions had 8 rare alleles and thirteen Indian accessions had 7 rare alleles. Among the non-Indian accessions Taiwan, Solo109 and Waimanalo had 2 while Kapoho and Taiwan Red Lady had 1 rare allele each. Among the Indian accessions Yellow Indian, Pusa Giant, Ranchi, Coorg Honey Dew, Surya, Madhu and RT2 each had 1 rare allele.

### Null alleles

As per the definition of null alleles by Callen et al. (1993) 19 null alleles were detected from 12 polymorphic loci. A null allele is shown in the gel image given in Figure 1. Average null alleles identified for the dinucleotide repeat amplifying markers was 0.79 alleles/marker and that for the trinucleotide repeat amplifying markers was 1.33 alleles/marker. The highest number of null alleles (3 alleles each) were identified in the profiles of SP2 and SSPA10 followed by SP5, SSPA7 and SSPA8 (2 alleles each). In this study no null alleles were detected in the 6 *Vasconcellea* and 1 *Jacaratia* accessions. The non-Indian *Carica papaya* accession from South Africa, Hortus Gold generated 5 null alleles. Waimanalo an accession from Hawaii and Taiwan Red Lady, a F1 hybrid of the Tainung series had 2 null alleles each. Another accession of the same series, Taiwan had one null allele. The Indian papaya accessions Yellow Indian, Coorg Honey Dew, Ranchi, Surya, Madhu, Orissa local, CO1, CO7 and PAU selection had one null allele each.

### Clustering of the Caricaceae accessions

**Figure 2:**
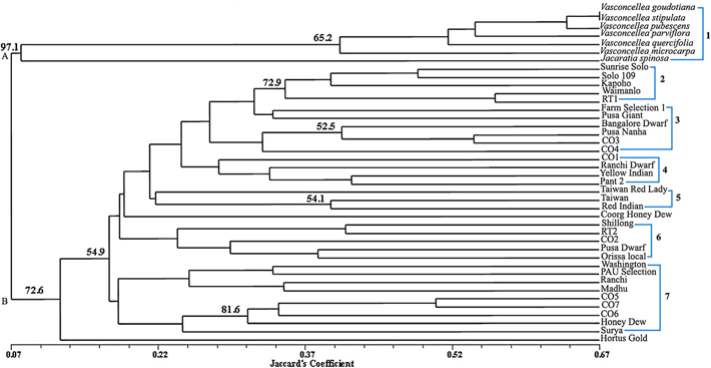
**Dendrogram of 41 Caricaceae accessions based on Jaccaard’s genetic similarity coefficient.**

The dendrogram given in Figure 2 was made from genetic similarity values. The strength of dendrogram nodes was estimated with a bootstrap analysis using 1000 permutations. The similarity among the Caricaceae accessions ranged from 7% to 67% and they were divided into 2 major clusters “A” and “B” and 7 sub clusters. At 7% level of similarity two distinct major clusters “A” and “B” were present. Major cluster “A” included 6 accessions of the 6 different *Vasconcellea* species along with the accession of the related species *Jacaratia spinosa.* Within this cluster, two accessions of *Vasconcellea goudotiana* and *Vasconcellea stipulata* were 67% similar amongst themselves. The second major cluster “B” consisted of the rest of the 34 *Carica papaya* accessions grouped into various clusters and sub clusters at higher levels of similarity.

Within major cluster “B”, at about 11.9% level of similarity, the South African accession Hortus Gold segregated out and the rest of the 33 Caricaceae accessions underwent segregation at 16% level of similarity and differentiated into sub clusters 2 to 7. Sub cluster 2 included the Hawaiian accessions of Sunrise Solo, Solo109, Kapoho and Waimanalo along with the Indian accession RT1. Among these the highest genetic similarity of about 54.5% was observed between the accessions Waimanalo and RT1. Sub cluster 3 included the accessions Farm Selection 1, Pusa Giant, Pusa Nanha, Bangalore Dwarf, CO3 and CO4. The highest genetic similarity of about 52% was observed between CO3 and CO4. Sub cluster 4 included the accessions CO1, Ranchi dwarf, Yellow India and Pant 2 with the highest genetic similarity of 38% between the last two accessions. Sub cluster 5 included the non-Indian accessions Taiwan Red Lady and Taiwan along with the Indian accession Red Indian. The highest genetic similarity of about 38% was observed between Taiwan and Red Indian. Sub cluster 6 included the Indian accessions Shillong, RT2, CO2, Pusa Dwarf, and Orissa Local with the highest genetic similarity of about 38% between Shillong and RT2. Sub cluster 7 included the Indian accessions Washington, PAU Selection, Ranchi, Madhu, CO5, CO6, CO7, Honey Dew and Surya. The highest genetic similarity of about 50% was observed between CO5 and CO7.

## Discussion

The inherent genetic diversity of this set of Caricaceae accessions was apparent from the analysis of the SSR profiles and from the dendrogram where all the accessions had unambiguously separated. In general all the SSR markers used in this study produced clear and consistent amplification profiles. Stutter bands, which were minor products amplified in PCR and had lower intensity than the main allele because they normally lacked or had extra repeat units; (Walsh et al. 1996) were also present in the profiles of most of the markers used. Rare alleles as described by Jain et al. (2004) was detected from within this set of accessions. Null alleles were present probably due to mutations in the binding region of one or both of the microsatellite primers, which inhibited primer annealing (Callen et al. 1993). The average number of SSR alleles for the Indian and non-Indian Carica papaya accessions were 6 alleles per locus. This number was much higher than that reported for average number of alleles by Oliveira et al. (3.18 alleles per locus, 2010a) and was much smaller than the study by Asudi et al. (2010) which reported 11.93 alleles/locus. Although this study was just an overview of the inherent genetic diversity, the Indian *Carica papaya* accessions produced more alleles per SSR marker (5.1 alleles/locus) as compared to those generated from the non-Indian *Carica papaya* accessions (3.5 alleles/locus) and the accessions of *Vasconcellea sp*. and *Jacaratia spinosa* (2.7 alleles/locus). Average PIC value was the highest among the Indian papaya accessions. However there was no significant difference of PIC values between the Indian and the non-Indian accessions. Twenty two out of the thirty seven rare alleles were detected in the seven accessions of *Vasconcellea sp and Jacaratia spinosa*. Therefore on an average 3.14 rare alleles per accession was detected in the former group as compared with 0.44 rare alleles per accession in case of the *Carica papaya* accessions. The accessions of *Jacaratia spinosa* and *Vasconcellea sp* segregated as a single cluster from the *Carica papaya* accessions with only 7% genetic similarity. Therefore in tune with the taxonomic descriptions of Badillo (2000), our findings also indicated that *Jacaratia spinosa* and *Vasconcellea sp* are distinctly different from *Carica papaya*. The same was reported by Van Droogenbroeck et al. (2002) while using Amplified Fragment Length Polymorphism (AFLP) to study genetic relationship among Caricaceae accessions. It was indicated by Droogenbroeck et al. (Van Droogenbroeck et al. 2002) that the *Carica papaya* accessions were very distinct from *Vasconcellea* on the basis of results of cluster analysis and bootstrap analysis. Moreover, they reported a higher level of similarity between *Vasconcellea* accessions and *Jacaratia spinosa*. Similar conclusion was also apparent from the dendrogram derived from our study. During the analysis of genetic diversity of *Carica papaya* using AFLP, Kim et al. (2002) found that *Carica papaya* shared the least genetic similarity with other Caricaceae accessions. The *Carica papaya* accessions from our study were also distant from the *Vasconcellea* and *Jacaratia* accessions. However the Indian accessions of *Carica papaya* in this study had more allelic diversity as per their SSR profiles. Among the 34 *Carica papaya* accessions, Hortus Gold a clone from South Africa with 5 null alleles was distinctly diverse from the rest of the 33 accessions having only 13% genetic similarity. Further insight into the allelic nature of other loci of this accession and its comparison with other *Carica papaya* accessions will make an interesting subject for examination of genetic diversity in future studies. Out of the 34 *Carica papaya* accessions, 19 were dioecious and 15 were gynodioecious. It was found during the study that the dioecious group of accessions shared amongst them a total of 101 alleles with an average of 5.05 alleles/marker. The gynodioecious group of accessions on the other hand had a total of 96 alleles amongst them with an average of 4.8 alleles/marker. The Indian accessions were mostly dioecious whereas all the non-Indian accessions were gynodioecious except for Hortus Gold, which was dioecious.

In the dendrogram the accessions Sunrise Solo, Solo 109, Kapoho and Waimanalo were grouped into the same sub cluster. In Hawaii, the parentage of most of the papaya cultivars was based on the cultivar Solo, which was introduced from Barbados and Jamaica in 1911. Solo gave rise to several varieties, like Sunrise, Sunset, Solo 109 and Kapoho. Sunrise Solo, one of the accessions in this study, was an inbred reddish orange strain resulting from a cross between “Pink Solo” with the yellow fleshed farmer’ selection “Kariya solo” (Hamilton and Ito, 1968). In the dendrogram these accessions were situated in close proximity. According to Ram (2005), the fruit morphology of Kapoho and Waimanalo was almost similar but genetic difference between them was high. As can be seen from the dendrogram, (Figure 2) Kapoho and Waimanalo was only about 38% similar genetically. Waimanalo was a genotype from Florida and was a selection from the cross between 5′ Solo and Betty.

In the same dendrogram (Figure 2) the accessions of Coimbatore varieties (CO1-CO7) were included in separate sub clusters. These accessions were developed in the South Indian state of Tamil Nadu and were bred in a span of 14 years by various workers and had distinct sex forms, fruit characteristics and papain yield. Principal genotype CO1 was a selection from the genotype Ranchi and CO2 was a selection from Peradeniya, a genotype from Sri Lanka. CO3 was developed from a cross between CO2 and Sunrise Solo and CO4 was the result of a cross between the genotypes CO1 and Washington. In the same manner the three accessions of the Pusa series; Pusa Nanha, Pusa Giant and Pusa Dwarf were also found in different sub clusters. Developed at IARI, New Delhi during 1966 to 1982 (Ram, 2005), Pusa Dwarf was an original selection from Pusa Giant and was very popular in Eastern Uttar Pradesh and Northern Bihar. Pusa Giant was selected to withstand high wind velocities in various parts of the country. Pusa Nanha was a dwarf mutant evolved by gamma irradiation (Ram and Sharma, 1996). Both Pusa Nanha and PusaDwarf have short plant height but differences in their origin had made them genetically diverse. The accessions pairs Ranchi, Ranchi Dwarf and Honey Dew, Coorg Honey Dew also segregated into different sub clusters. Ranchi was an accession found in the state of Jharkhand, India. It has a large number of segregating populations which were found in Northern India. Ranchi Dwarf was a selection from Ranchi. Honey Dew was bred in the South Indian state of Karnataka and Coorg Honey Dew was a selection done at Chethalli, Coorg in 1959. According to Ram (2005) both Ranchi and Honey Dew are “Minor genotypes” i.e. plants grown from seeds of selected fruits of the traditional cultivars. Both Ranchi Dwarf and Coorg Honey Dew were categorized by Ram (2005) as being “Local adaptive genotype” which was described as phenotypic selection having better plant type, quality and productivity from within the minor genotypes. The “Local adaptive genotypes” are more suitable for growing in special agro-climatic zones. As was evident from the dendrogram this adaptation caused enough genotypic modifications so as to segregate the accessions into different clusters.

## Conclusions

In view of the results obtained it was perceptible that, at least for the loci amplified by the markers and for the set of accessions used, the Indian *Carica papaya* accessions had more allelic diversity than the non-Indian cultivars and the Caricaceae species included in this study. However there was no significant difference of average PIC values between the Indian and the non-Indian accessions. Although *Vasconcellea* and *Jacaratia* are distant from *Carica papaya* in the evolutionary perspective yet there must be some genotypic and phenotypic similarity as they had been classified under the same family, Caricaceae. At least in case of the loci amplified by the 20 markers used in this study, may be such kind of similarity had been reflected hence no null alleles were produced but some rare alleles have been manifested. The Indian *Carica papaya* accessions have a long history of domestication and were bred by various plant breeders to suit the varied Indian agro-climatic conditions (Ram, 2005). Within the Indian territory these accessions had undergone indiscriminate cross–pollination along with high degree of wind and insect pollination up to a distance of several kilometers leading to the production of numerous local mixtures everywhere in the country (Prest, 1955). They also had diverse parents which include genotypes from within the country as well as genotypes from South East Asia and North America (Ram, 2005). This variation combined with spontaneous mutations had perhaps made the genetic base for the Indian genotypes much wider than the non-Indian *Carica papaya* accessions and the accessions of *Vasconcellea sp*. and *Jacaratia spinosa* included in this study. Off course further insight into the inherent genetic diversity of these and other Indian papaya accessions and their comparisons to a larger number of non-Indian accessions is required to form a decisive statement on the genetic nature of Indian *Carica papaya* accessions.

## Materials and methods

### Plant materials

The germplasm set in this study included 1 accession each from 27 Indian and 7 non-Indian commercially popular *Carica papaya* cultivars, 1 accession each of *Vasconcellea goudotiana*, *Vasconcellea microcarpa*, *Vasconcellea parviflora*, *Vasconcellea pubescens*, *Vasconcellea stipulata* and *Vasconcellea quercifolia* and 1 accession of South American tree species *Jacaratia spinosa.* The collection was maintained at the experimental farm of Acharya J.C. Bose Biotechnology Innovation Centre, Bose Institute at Madhyamgram. Fully expanded fourth leaf from the top was used as plant material for genomic DNA isolation. The category, cultivar name, source and number of accessions use in this study for each cultivar are given in Table 3.

**Table 3:** **Category, cultivar name, source and number of accessions used for this study**

Indian ***Carica papaya*** cultivars				
Category Ram (2005)	Cultivar name	Sex form	Source	Number of accessions
Local adaptive genotype	Ambasa Local (RT2)	Dioecious	ICAR, Tripura	1
Local adaptive genotype	Bangalore Dwarf	Dioecious	Pvt. seed company	1
Local adaptive genotype	Coorg Honey Dew	Gynodioecious	ICAR, Tripura	1
Local adaptive genotype	Farm Selection -1	Gynodioecious	Pvt. seed company	1
Local adaptive genotype	Madhu	Gynodioecious	Pvt. Seed company	1
Local adaptive genotype	Orissa local	Dioecious	OUAT	1
Local adaptive genotype	Pant 2	Dioecious	IIHR	1
Local adaptive genotype	PAU Selection	Dioecious	IIHR	1
Local adaptive genotype	Ranch Dwarf	Dioecious	Pvt. Seed company	1
Local adaptive genotype	Shillong	Dioecious	IIHR	1
Local adaptive genotype	RT1	Dioecious	ICAR, Tripura	1
Local adaptive genotype	Washington	Dioecious	IIHR	1
Minor genotype	Honey Dew	Gynodioecious	ICAR, Tripura	1
Minor genotype	Ranchi	Dioecious	Pvt. seed company	1
Principal genotype	CO 1	Dioecious	ICAR, Tripura	1
Principal genotype	CO 2	Dioecious	TNAU	1
Principal genotype	CO 3	Gynodioecious	TNAU	1
Principal genotype	CO 4	Dioecious	TNAU	1
Principal genotype	CO 5	Dioecious	TNAU	1
Principal genotype	CO 6	Dioecious	Pvt. seed company	1
Principal genotype	CO 7	Gynodioecious	TNAU	1
Principal genotype	Pusa Dwarf	Dioecious	IIHR	1
Principal genotype	Pusa Giant	Dioecious	IIHR	1
Principal genotype	Pusa Nanha	Dioecious	IIHR	1
Principal genotype	Red Indian	Gynodioecious	Pvt. seed company	1
Principal genotype	Surya	Gynodioecious	IIHR	1
Principal genotype	Yellow India	Gynodioecious	Pvt. seed company	1
**Non-Indian*Carica papaya*cultivars**				
**Category**	**Accession name**		**Source**	**Number of accessions**
South African cultivar	Hortus Gold	Dioecious	Pvt. seed company	1
Hawaiian cultivar	Kapoho	Gynodioecious	USDA	1
Hawaiian cultivar	Solo Papaya 109	Gynodioecious	USDA	1
Hawaiian cultivar	Sunrise Solo	Gynodioecious	USDA	1
F1 hybrid Tainung series	Taiwan	Gynodioecious	Pvt. seed company	1
F1 hybrid Tainung series	Taiwan Red lady	Gynodioecious	USDA	1
American cultivar (Florida)	Waimanalo	Gynodioecious	Pvt. seed company	1
**Other*Caricaceae*species**				
**Category**	**Accession name**		**Source**	**Number of accessions**
Highland papaya	*Vasconcellea goudotiana*	Dioecious	USDA	1
Highland papaya	*Vasconcellea microcarpa*	Dioecious	USDA	1
Highland papaya	*Vasconcellea parviflora*	Dioecious	USDA	1
Highland papaya	*Vasconcellea pubescens*	Dioecious	USDA	1
Highland papaya	*Vasconcellea stipulata*	Dioecious	USDA	1
Highland papaya	*Vasconcellea quercifolia*	Dioecious	USDA	1
Related genus	*Jacaratia spinosa*	Dioecious	USDA	1

*ICAR:* Indian Council of Agricultural Research, *IIHR:* Indian institute of Horticultural Research, *OUAT:* Orissa University of Agriculture and Technology, *TNAU:* Tamil Nadu Agriculture University, *USDA:* United States Department of Agriculture.

### Isolation of genomic DNA and PCR amplification

Genomic DNA isolation was done according to the method of Walbot (1988). PCR amplification of this DNA was done with 20 SSR markers. Ten of the markers used were designed by Chun et al. (2006) and the rest by Eustice et al. (2008), were used to study the genetic diversity. The name, BAC-End sequence name, motif, reference, forward and reverse primer and annealing temperature of those markers are given in Table 4. DNA amplification was carried out in 25 μl volumes using 200 μl thin-walled PCR tubes (Axygen, USA) in a MJR thermal cycler. Each reaction mixture contained 1 μl of genomic DNA (100 ng), 0.5 μl of each of the two primers (at a concentration of 10 pmole/μl), 2.5 μl of a 10X PCR buffer, 0.75 μl of a 50 mM MgCl_2_ solution, 0.25 μl of a 2.5mM dNTP mixture, 0.2 μl (1 unit) of a 5 unit/μl Taq DNA polymerase and 19.3 μl of PCR-grade water. The temperature profile used for PCR amplification was 97°C for 5 mins, 55-60°C (as necessary in accordance to Table 4) for 2 min; followed by 35 cycles of 1 min at 95°C, 1 min at 55-60°C and 2 min at 72°C. The final extension was at 72°C for 10 min.

**Table 4:** **Name, BAC-End sequence name, motif, reference, forward and reverse primer and annealing temperature of the SSR markers used**

Name	BAC-End sequence name	Motif	Reference	Forward Primer	Reverse Primer	TºC
**SP 1**	Pbac -102C-06.T1.G06	(TTTC)5/(TTC)9	Chun et al., (2006)	TGCAACAGAAATAAAAACAGCA	GACGTGGACGAGCTCTGTGT	51
**SP 3**	Pbac -14D-B01.t1_009.ab1	(AC)_9_	Chun et al., (2006)	CACCAACAAGTTCCTTGGGT	TGCATGCATGTGTGTGGATA	57
**SP4**	Pbac -15C-B06.t1.B06.ab1	(AT)_9_	Chun et al., (2006)	TGCTCATAAAGTGATGGAGGT	TGGCGACCATTTAAACAACA	55.5
**SP5**	Pbac -15C-E11.y1.E11.ab1	(AC)_9_	Chun et al., (2006)	TTGGCTTCAAATTCAGGCTT	GCGGCTTCTGGATCTGATAA	56
**SP6**	Pbac -16C-C10.y1.C10.ab1	(AT)_9_	Chun et al., (2006)	CTTGCACCGAACCCTAAAAG	CATGAAAAACACATGCCTGC	57
**SP7**	Pbac -16A-B08.y1_063.ab1	(AAT)_7_	Chun et al., (2006)	CAGTTGTAGGGGTTGGTGGT	GTCCACAAATCAGAGCCCAT	59
**SP8**	Pbac -2B-F07.Y1_061	(ATT)7	Chun et al., (2006)	CAAATCATGTTGGTCTGCGT	GCTCAGCGGCTATTTTTGAC	57
**SP9**	Pbac -28D-E10.t1_081.ab1	(AC)10	Chun et al., (2006)	TCAATGAGCCCCTCAATTTC	ATGGATGGATTCAGCCGTTA	56
**SP10**	Pbac -3C-H06.t1_057.ab1	(CT)20	Chun et al., (2006)	CGACGTCGTTTTCTCCTTTC	CACACATCGTGGGTTGAAGT	58
**SSPA 1**	Pbac-102A-H04.Y1.h04.scf	(A)21	Eustice et al., (2008)	TCATCGTCTTCAACCTGTAGC	ATCGACCTCCTCCATCACAC	61
**SSPA 2**	Pbac-102C-01.T1.H01.scf	(AT)12	Eustice et al., (2008)	ACCAGAGTGGACCCAGTAGC	TGTTCACGTAAGGCATCCTG	61
**SSPA 3**	Pbac-102D-A11.T1.A11.scf	(AG)10	Eustice et al., (2008)	CGAAGCAAAACTTCTCAGCC	TCTCAATTTCCATTTTCCGC	58
**SSPA 4**	Pbac-10B-F10.T1_089.ab1	(TTC)10	Eustice et al., (2008)	GTGCAAGTCTCTCGAGTCCC	CTTGCTTTGCACTTTTCAGG	61
**SSPA 5**	Pbac-10C-c04.y1_023.ab1	(TA)11	Eustice et al., (2008)	CACGAACAACTGTCACCCAC	TCAAGACCTTTGCATGATGG	61
**SSPA 6**	Pbac-10C-G06.t1_049.ab1	(TA)12	Eustice et al., (2008)	GCTGCATCGACATTTACGAA	TCAAGCCTGAGGAATCTGCT	59
**SSPA 7**	Pbac-1D-F03.y1_028.ab1	(AG)24	Eustice et al., (2008)	TTCAAATCTTTTTCGCACCC	TCAACAGCTTCGTTGACCAG	59
**SSPA 8**	Pbac-21C-F01.t1F01.scf	(AT)12	Eustice et al., (2008)	TGTCTCAGCATATCCACCCA	ATGGCCTTTTGGAACATCAG	60
**SSPA 9**	Pbac-25ZB-F01.y1_012.ab1	(AAG)10	Eustice et al., (2008)	GCAGAAGCCAACAGCTCTCT	AGATCTAGCAGCCGCCATAA	61
**SSPA 10**	Pbac-28A-B03.t1_026.ab1	(TTC)7	Eustice et al., (2008)	AGGAATGCCCTCCATGTAAA	AGGAATGCCCTCCATGTAAA	59
**SP 3**	Pbac -14D-B01.t1_009.ab1	(AC)_9_	Chun et al., (2006)	CACCAACAAGTTCCTTGGGT	TGCATGCATGTGTGTGGATA	57

Name – Identifiers given to each marker in our laboratory TºC – Annealing temperature as determined during our experimentation.

### Polyacrylamide Gel electrophoresis

The PCR products were resolved by native polyacrylamide gel electrophoresis (PAGE) following the protocol given by Sambrook et al. (1989) in a 6% gel in vertical electrophoresis tank (gel size of 16 cm × 14 cm, Biotech, India) with Tris-Acetate-EDTA buffer at 150V supplied by a power pack. The gel, after electrophoresis, was stained with ethidium bromide (5 μg of EtBr in 200 ml of Tris-Borate-EDTA buffer) washed thoroughly with double distilled water and photographed using a Gel Documentation System (Biorad, USA).

### Allele scoring

Under UV light a cluster of 2 to 5 discrete bands (stutter) was apparent in the stained gels for most of the markers. The size (in nucleotides) of the most intensely amplified band for each microsatellite marker was determined using the software Quantity One (Biorad, USA), based on the migration of the band relative to molecular weight size markers (100 bp DNA ladder SibEnzyme) included in the gel (Cho et al. 2000). The molecular weights of the Carica papaya cultivar SunUp, as derived from previous experiments (Papaya BAC end sequence library developed by the Hawaii Agricultural Research Center and the Center for Genomcis, Proteomics, and Bioinformatics Research Initiative) was used as a molecular weight reference for each marker. The band with the lowest molecular weight for each SSR marker was assigned allele number 1 and the progressively heavier bands were assigned incrementally. For the individual markers, the presence of an allele in each of the germplasms was recorded as ″1″ and the absence of an allele was denoted as ″0″ (Cho et al. 2000). Null alleles were assigned when no amplification product was generated (Callen et al. 1993). When an allele was found in less than 5% of the germplasms under study, it was designated as rare (Jain et al. 2004).

### Genetic diversity analysis using SSR profiles

A 1/0 matrix was constructed for each marker using the information of presence or absence of alleles and was used to calculate genetic similarities among the accessions according to Jaccard (1908) using NTSYS-pc software package (version 2.02e) (Rohlf 1997). Using pairwise similarity matrix of Jaccard’s coefficient, a phylogenetic tree was made by unweighted pair-group method of arithmetic average (UPGMA) and neighbor-joining (NJoin) module of the NTSYS-pc. Support for clusters was evaluated by bootstrap analysis using WinBoot software (Yap and Nelson 1995) through generating 1,000 samples by re-sampling with replacement of characters within the combined 1/0 data matrix. The average polymorphic information content (PIC) was calculated for each marker in accordance with the method Anderson et al. (1997).

### One way ANOVA

One way ANOVA was done to test whether the means of the PIC values for the total set of germplasms, and for the three categories (Indian and non Indian *Carica papaya* accessions and the accessions of *Vasconcellea* species and *Jacaratia spinosa*) were significantly different or not. The software SPSS 10.0 was used for the purpose.

## Authors’ information

SS – Senior Research Fellow, Department of Horticulture, Institute of Agricultural Science, University of Calcutta, 35, Balligunge Circular Road, Kolkata 700029, West Bengal, India.

BD – Senior Research Fellow, Division of Plant Biology, Bose Institute, Main Campus, 93/1 A.P.C. Road, Kolkata 700009, West Bengal, India.

MP – Scientist National Institute of Plant Genome Research (NIPGR), Aruna Asaf Ali Marg, New Delhi 110067, India

PA – Assistant Professor, Department of Horticulture, Institute of Agricultural Science, University of Calcutta, 35, Balligunge Circular Road, Kolkata 700029, West Bengal, India.

TKG – Associate Professor, Division of Plant Biology, Bose Institute, Main Campus, 93/1 A.P.C. Road, Kolkata 700009, West Bengal, India.
